# An experimental platform using high-power, high-intensity optical lasers with the hard X-ray free-electron laser at SACLA^1^ ^1^

**DOI:** 10.1107/S1600577519000882

**Published:** 2019-02-22

**Authors:** Toshinori Yabuuchi, Akira Kon, Yuichi Inubushi, Tadashi Togahi, Keiichi Sueda, Toshiro Itoga, Kyo Nakajima, Hideaki Habara, Ryosuke Kodama, Hiromitsu Tomizawa, Makina Yabashi

**Affiliations:** a RIKEN SPring-8 Center, 1-1-1 Kouto, Sayo, Hyogo 679-5148, Japan; b Japan Synchrotoron Radiation Research Institute, 1-1-1 Kouto, Sayo, Hyogo 679-5198, Japan; c Graduate School of Engineering, 2-1 Yamada-oka, Suita, Osaka 565-0871, Japan; d Institute of Laser Engineering, Osaka University, 2-6 Yamada-oka, Suita, Osaka 565-0871, Japan

**Keywords:** X-ray free-electron laser, high-intensity optical laser, high-energy density science, SACLA

## Abstract

An experimental platform using X-ray free-electron laser (XFEL) pulses with high-intensity optical laser pulses is open for early users’ experiments at the SACLA XFEL facility after completion of the commissioning. The combination of the hard XFEL and the high-intensity laser provides capabilities to open new frontiers of laser-based high-energy-density science. During the commissioning phase, characterization of the XFEL and the laser at the platform has been carried out for the combinative utilization as well as the development of instruments and basic diagnostics for user experiments. An overview of the commissioning and the current capabilities of the experimental platform is presented.

## Abstract 2

An experimental platform using X-ray free-electron laser (XFEL) pulses with high-intensity optical laser pulses has been developed at the SACLA XFEL facility. An overview of the commissioning and the capabilities of the experimental platform is presented.

## 1. Introduction   

X-ray free-electron lasers (XFELs) (Emma *et al.*, 2010 ▸; Ishikawa *et al.*, 2012 ▸) have had a significant impact in broad scientific areas because of their unique properties: high brilliance, short pulse-duration and high spatial coherence. Laser-based high-energy-density (HED) science is one of the promising research topics for which XFELs provide new experimental approaches to crucial scientific questions that need to be answered.

High-power lasers, including short-pulse femtosecond lasers and long-pulse nanosecond lasers, have been employed in various scientific and engineering research such as particle acceleration, high-pressure excitation, astrophysics, hot/warm dense matter (HDM/WDM) production and fusion energies (Leemans *et al.*, 2006 ▸; Denoeud *et al.*, 2016 ▸; Kraus *et al.*, 2016 ▸; Albertazzi *et al.*, 2017 ▸; Millot *et al.*, 2015 ▸; Remington, 2005 ▸; Ogitsu *et al.*, 2012 ▸; Ping *et al.*, 2010 ▸; Kodama *et al.*, 2002 ▸; Ditmire *et al.*, 2004 ▸). In recent years, high-power lasers with focused intensities in the relativistic regime (>2 × 10^18^ W cm^−2^ for 800 nm wavelength) have been commercially available with a short pulse duration of the order of tens of femtoseconds at relatively high repetition rates (∼1 Hz or more) (Danson *et al.*, 2015 ▸). Matter irradiated with such high-intensity laser pulses is heated to high temperature immediately and becomes plasmas, which are strongly non-equilibrium states in space and time. It is important to understand the laser–matter interactions and phenomena thereafter in the laser-irradiated matter for scientific and engineering applications using high-intensity lasers. Various probing techniques have been developed and applied for this purpose not only with an optical probe but also with laser-produced X-rays or particles (electrons, ions and neutrons) (Park *et al.*, 2004 ▸; Glenzer & Redmer, 2009 ▸; Schumaker *et al.*, 2013 ▸; Borghesi *et al.*, 2002 ▸; Li *et al.*, 2010 ▸). Here, the challenges of experimental research to address the questions are due to the extreme temporal and spatial scale of objects and high plasma density. In comparison with traditional probes, the XFEL has great advantages as a probe: (i) an ultimate temporal resolution because of its ultra-short pulse duration, (ii) a fine spatial resolution because of its short wavelength and coherency, (iii) an efficient penetration into solid-density plasma because of its high critical density (Kluge *et al.*, 2018 ▸).

Moreover, the XFEL itself can be a unique candidate as a pump to produce matter with HED states (Vinko *et al.*, 2012 ▸; Hau-Riege *et al.*, 2012 ▸; Zastrau *et al.*, 2014 ▸; Yoneda *et al.*, 2014 ▸; Rackstraw *et al.*, 2015 ▸). Since the XFEL is a coherent light source, the full beam of the pulse can be focused down to nanometres and achieve focused intensities of 10^20^ W cm^−2^. Assuming an XFEL pulse focused to a micrometre spot with a pulse energy of a few hundreds of micro-joules absorbed in matter with an attenuation length of a few micrometres (for the case that the X-ray photon energy is just above the *K*-edge, for example), the energy density can be of the order of 10^13^ J m^−3^, which fulfills the condition of the state of ‘high energy density’ (>10^11^ J m^−3^). When the photon energy is optimized for the material of the samples, the XFEL can heat the matter deeply with a small temperature gradient in the longitudinal direction, which is difficult with an optical laser since the laser deposits its energy on the surface of the matter. Therefore, in addition to the unique energy deposition processes of X-rays in matter, *i.e.* the direct ionization of inner-shell electrons, the HED states or the WDM produced by the XFEL can be research objects for the further understanding of HED science.

Since the XFELs are strongly expected to expand the research capabilities of HED science as described above, there has been a high demand for an experimental platform where XFELs can be used in combination with high-intensity lasers. The development of an experimental platform that meets with this demand has been proposed at the world’s first compact XFEL facility, SACLA, similar to other XFEL facilities including the Linac Coherent Light Source (LCLS) (Nagler *et al.*, 2015 ▸; Glenzer *et al.*, 2016 ▸) and the European XFEL (Nakatsutsumi *et al.*, 2017 ▸). The commissioning of the platform at SACLA has been completed and early users’ experiments started in 2018.

In this paper, we describe an overview of the commissioning and the current status of the experimental platform with high-intensity lasers at SACLA.

## 2. Experimental platform   

### 2.1. Overview   

**Figure 1:**
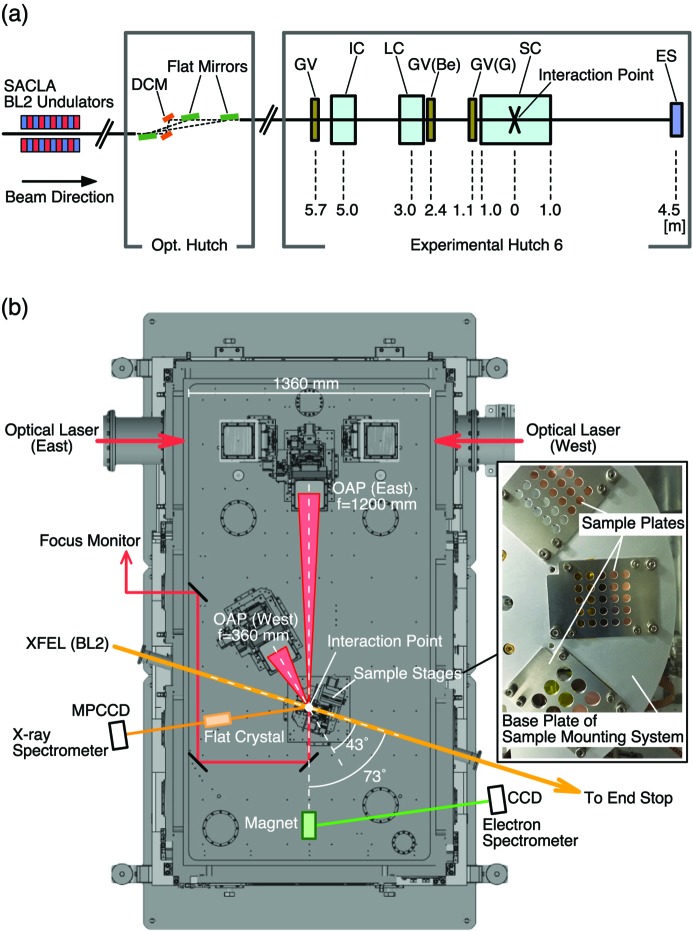
Overview of the experimental platform. (*a*) Basic optics for the XFEL beam transport in the OH and beamline components in EH6. The interaction point in the sample chamber (SC) is 5.7 m from a vacuum gate valve (GV) at the entrance of EH6. The beam monitors and CRLs are in an instrument chamber (IC) and a lens chamber (LC), respectively. The CRLs are isolated from the SC either with the GV with a glass (G) or beryllium (Be) window. The end stop of the beam is 4.5 m from the interaction point. (*b*) Schematic setup in the sample chamber (top view of the lower floor) for the commissioning experiments. The optical path for the west laser beam is shown for future reference. The inset shows an example of sample plates mounted on the sample mounting system.

The experimental platform for the combinative use of the XFEL and the high-intensity lasers is located in experimental hutch 6 (EH6) at the end of the second hard X-ray beamline (BL2) of SACLA in the SACLA SPring-8 experimental facility (Fig. 1 ▸). The Ti:sapphire laser system (800 nm wavelength) for this platform is designed to deliver two beams at a repetition rate of 1 Hz with a maximum energy of 12.5 J in a pulse duration of 25 fs in each beam, which derives a peak power of 500 TW after pulse compression. The three lasers (one X-ray and two optical lasers) can be used simultaneously in a vacuum chamber for pump–probe experiments in a variety of combinations, for example, ‘pumped with optical laser and probed with XFEL’, ‘pumped with laser-produced particles and probed with XFEL’ and ‘pumped with XFEL and probed with laser-produced particles’. For the radiation shielding from the energetic particles produced by the high-intensity lasers, the structure of EH6 contains a thicker radiation shielding compared with the other experimental hutches at SACLA. Furthermore, additional radiation monitors are equipped and integrated into the laser interlock system. The allocation at the last experimental hutch of the beamline allows installation of the large vacuum chamber (1.4 m × 2.7 m × 1.6 m) and related components for experiments permanently on the beamline without any interference with other experiments performed at the beamline. The sample chamber consists of two floors. The high-intensity lasers are transported on the upper floor and then delivered to the lower floor for focusing and irradiation to the sample, where the XFEL passes through. The two breadboards (one breadboard for each floor) in the sample chamber are isolated from the outer walls of the chamber to minimize the influence of the vibration and the deformation of the walls under vacuum conditions.

### 2.2. Light source performance of XFEL   

Two hard XFEL beamlines (BL2 and BL3) are operational for user experiments at SACLA by sharing the common accelerators with electron energies up to 8.5 GeV. General information about BL2 has been given by Tono & Hara (2017 ▸) and Yabashi *et al.* (2017 ▸). At BL2, photon energies in the range 4–15 keV are used for experiments. The typical XFEL spectrum bandwidth is 0.5%. The pulse can contain energy over 500–600 µJ within a pulse duration of <10 fs. The undulators of BL2 are off the accelerator axis; therefore the electron beam needs to be bent by a kicker magnet and transported with twin double-bend achromat optics to cancel the beam distortion and fluctuations due to the coherent synchrotron radiation (Hara *et al.*, 2018 ▸). Note that the kicker magnet and its power supplies are operational for the beam at a 60 Hz repetition rate with energies between 5 and 8 GeV (Kondo *et al.*, 2018 ▸). Since the electron beam energy can be controlled on a pulse-to-pulse basis (Hara *et al.*, 2013 ▸), a system with variable-gap undulators can provide XFELs to the two beamlines (BL2 and BL3) pulse-to-pulse with different photon energies. The electron pulses are currently split into the two beamlines equally (*i.e.* 30 Hz each for 60 Hz operation). Arbitral switching is planned to increase the efficiency of the facility operation, particularly in pump–probe experiments performed with a low-repetition-rate laser such as the one in EH6.

The XFEL beam produced in the BL2 undulators is delivered to experimental hutches after a front-end section and an optics hutch (OH) where basic beamline optics and beam diagnostics are installed (Tono *et al.*, 2013 ▸, 2019 ▸). Either a set of plane mirrors or a double-crystal monochromator (DCM) (Ohashi *et al.*, 2013 ▸) is used to deliver beam downstream with a full or a limited spectral width of the incoming XFEL. There are two sets of plane mirrors of uncoated silicon for the beam transport with photon energies below 7.5 keV or 15 keV. The glancing angles of the mirrors are 4 mrad and 2 mrad, respectively. The DCM using Si(111) crystals can be used for photon energies from 4 to 30 keV to produce a monochromatic beam with a bandwidth of the order of 10^−4^. The plane mirrors and the DCM are designed to change the beam height by 20 mm in any case for γ-ray shielding.

**Figure 2:**
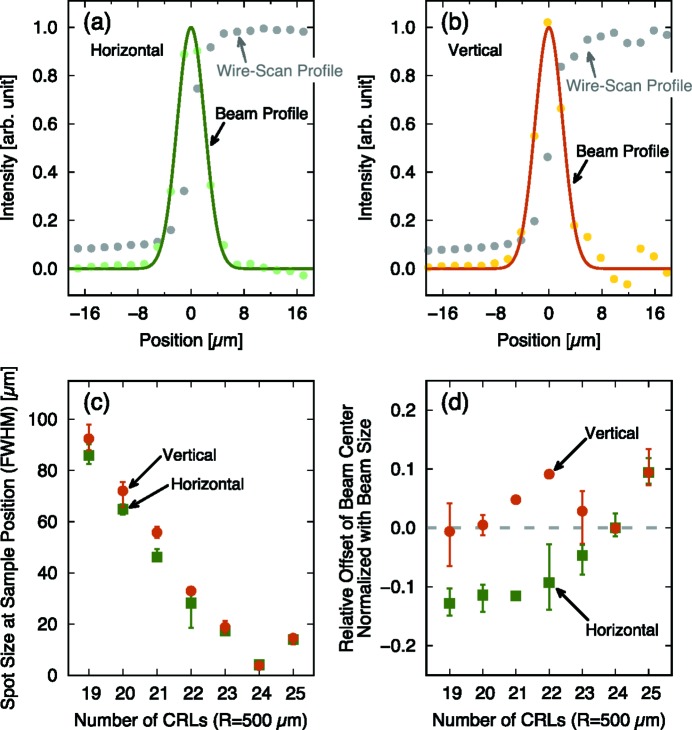
Demonstrated performance of the XFEL focus at 10 keV photon energy. Examples of focused profiles measured with the wire-scan technique at the sample position in (*a*) the horizontal and (*b*) the vertical directions, respectively, using 24 layers of *R* = 500 µm lenses. The spot sizes are 4–5 µm FWHM. (*c*) Spot-size dependences on the number of CRLs with radii of curvature of 500 µm. (*d*) Offsets of the focused beam position relative to the case with 24 layers of CRLs. The offsets are shown normalized with the beam size. Error bars in (*c*) and (*d*) represent scan-to-scan fluctuations.

In EH6, basic instruments are equipped to monitor the spatial profile and the pulse energy of the XFEL beam, similar to the other experimental hutches. Sets of compound refractive lenses (CRLs) are installed for the XFEL focusing in EH6 since the CRLs are on-axis optics which do not change the beam direction. This is an important advantage for the experimental platform with large laser systems to fix the sample position, the laser optics and the instruments independently of the XFEL focusing conditions. Here, the CRLs are made of beryllium with a rotationally parabolic structure in two dimensions (2D) with a geometric aperture larger than 1 mm. CRLs with various radii of curvature (*R*) at the vertex of the parabolic lenses are equipped in the system; *R* = 500, 1000, 1500 and 2000 µm. The sets of CRLs are placed on or displaced from the XFEL axis using air cylinders with a high repositioning accuracy; therefore, the selection of layers and curvatures of CRLs can be quickly controlled from outside of EH6. The maximum number of CRLs with *R* = 500 µm is 63, which is enough to focus the X-rays with energies from 4 to 15 keV at the sample position. Note that the distance from the center of the CRL system to the sample is 3 m [Fig. 1(*a*) ▸]. The sample position is ∼140 m away from the end of the undulators. The focusing capability for a 10 keV XFEL has been demonstrated as shown in Fig. 2 ▸. Here, the spot of the XFEL is measured by wire-scan technique using a gold wire. The minimum beam size measured at the sample position is about 3 µm full width at half-maximum (FWHM). It is also shown that the offset of the beam pointing is kept to less than ∼15% of the spot size when the spot size is changed from a few micrometres to about 100 µm [Figs. 2(*c*) and 2(*d*) ▸]. After passing through the sample position, the XFEL pulses exit from the sample chamber through either a beryllium window or a Kapton window (ICF 114), which is ∼1 m away from the sample. There is a beam stop at ∼4.5 m from the sample position.

### 2.3. Light source performance of the high-intensity laser   

**Figure 3:**
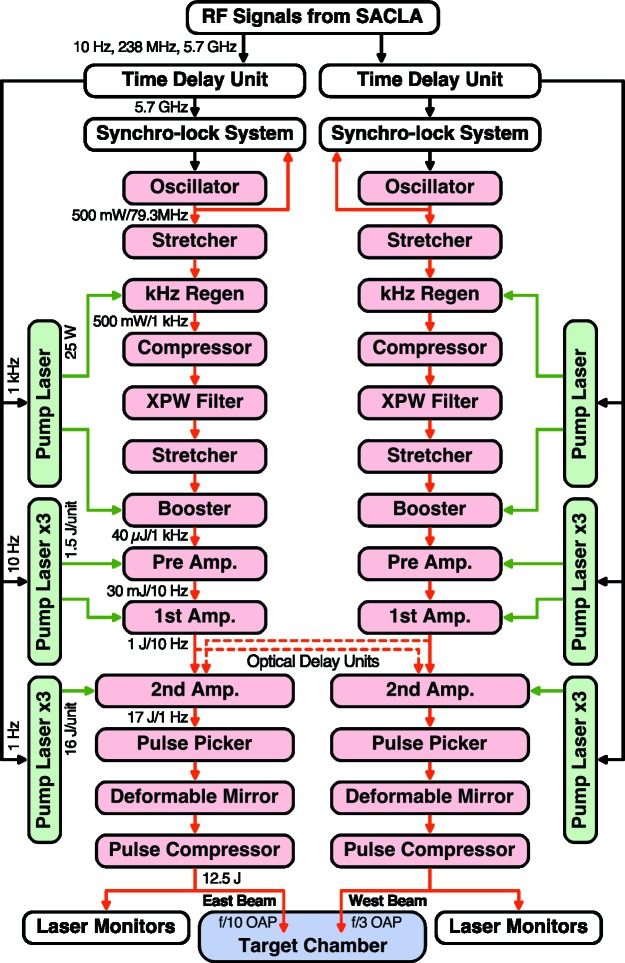
Overview of the high-intensity optical laser system. The system consists of two identical beamlines. Either of the frontend systems (from the oscillator to the first amplifier) can be shared between two beams as indicated by dashed arrows. An optical delay unit is installed in the path from the first amplifier to the second amplifier to compensate for the difference of the optical paths in the shared frontend mode. The east beam has been utilized in the early users’ experiments.

The laser system has been installed in a clean room, laser hutch 6 (LH6), separated from the experimental hutch. Major power supplies and electronic components of the laser systems are placed out of LH6 to stabilize the temperature of the laser room. The pulse compressors and laser monitors are located between LH6 and EH6 (outside of the radiation shielding), therefore, easily accessed during experiments if necessary. An overview of the laser system is shown in Fig. 3 ▸.

The laser system is constructed to produce two laser beams in two operation modes: ‘independent frontend mode’ and ‘shared frontend mode’. In the independent mode, two beams are produced independently from two sets of identical laser systems. In the shared frontend mode, the optical components before the second amplifiers are shared by the two beams. In this mode, a laser beam is split into two beams after the frontend, then delivered to the final amplifiers separately. The operation modes will be selected based on the requirements for the experiments. In the former case, the requirements on system alignment are relaxed to achieve the best performance of each beam. However, the timing jitter between two beams can be an issue for experiments requiring fine-timing adjustments. In the latter case, the timing-jitter between two beams can be minimized. However, more precise alignment is required to optimize both beams simultaneously and to achieve the required energy, because many optical components are shared in the frontend so that limited parameters are tunable for each beam.

In either operation mode the oscillator is synchronized to the XFELs using radiofrequency (RF) signals from SACLA as a timing signal (clock). The RF clock signal with a frequency of 5.7 GHz is delivered to the synchro-lock system through a time delay unit. The synchro-lock system equipped with a balanced optical-microwave phase detector (BOM-PD) (Kim *et al.*, 2004 ▸, 2006 ▸) monitors the phase balance between the 5.7 GHz RF signal and the 79.3 MHz laser pulses from the oscillator. Then, the system controls the fast and slow piezo actuators in the oscillator to adjust its cavity length for the further synchronization of the laser to the RF signal. The laser timing relative to the RF clock, and therefore to the XFEL, can be changed with the time delay unit for adjusting the RF clock signal.

In the laser system, the temporal contrast is improved via the cross-polarized wave generation (XPW) filter (Jullien *et al.*, 2005 ▸) and also the wavefront is corrected by a deformable mirror before the pulse compression. The laser monitors are equipped to diagnose the laser properties after the pulse compression, such as a wavefront sensor, an optical spectrometer, a beam pointing monitor, pulse duration and contrast monitors, and near-/far-field monitors. A cross-calibrated energy meter is installed, which detects the laser light through a mirror before the pulse compression, to measure the laser energy on a single-shot basis during the experiments.

**Figure 4:**
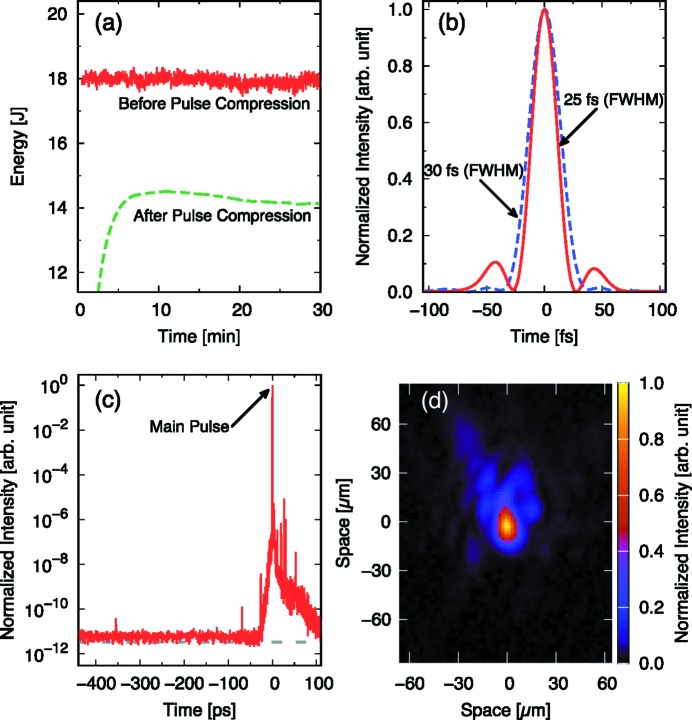
Observed characteristics of the high-intensity laser during the commissioning. (*a*) Laser pulse energies before (solid line) and after (dashed line) the pulse compression. The pulse energies are measured before the pulse compression at 1 Hz repetition rate with the cross-calibrated energy meter. A power meter detects the compressed pulses (30 fs duration) in the vacuum with a slow response. (*b*) Typical pulse profiles after the compression with durations of 25 fs and 30 fs FWHM. (*c*) Typical temporal contrast of the compressed pulse (∼40 fs). The dashed line indicates the noise level of the detector. (*d*) Example of a focus spot profile at the sample position.

One beam, the ‘east beam’, of the high-intensity laser system has been commissioned and operated for user experiments. Since a single beam is provided for experiments currently, the laser system is operated in independent frontend mode. During the commissioning phase, the laser pulse has been characterized, particularly the key properties that directly affect the experimental results: the energy stability, the pulse shape and the temporal contrast. The energy stability has been measured for the fully amplified pulses as shown in Fig. 4(*a*) ▸. In the measurements, the chirped laser pulses with energy of ∼18 J are compressed to ∼30 fs at 1 Hz repetition rate. Note that the pulse energy before the compression has been recorded for each shot; however, the energy of a single pulse after the compression has not been measured due to the slow response of the detector used in the vacuum (the response time is ∼10 min). The energy fluctuation before the pulse compression is 0.14 J or 0.76% in r.m.s. for 30 min (1800 shots). The compressor efficiency is estimated to be ∼78%. The optimization of the pulse compression has also been performed with the fully amplified pulses. However, in this case the pulse energy was attenuated using low-reflection optics after the final amplification to adjust the energy delivered to the monitors and also to avoid any damage to optics in the compression chamber. As one can see from Fig. 4(*b*) ▸, which shows the typical pulse profiles measured with the self-referenced spectral interferometry technique (Moulet *et al.*, 2010 ▸), the pulse with 25 fs duration in FWHM contains extra components before and after the main pulse. Such components are significantly suppressed when the pulse duration is adjusted slightly longer, ∼30 fs in FWHM. The temporal contrast has been measured for ∼100 mJ pulses from the frontend based on a third-order cross-correlation technique with a dynamic range above 10^11^ [Fig. 4(*c*) ▸]. The contrast is better than 10^10^ until 30 ps before the main pulse.

The east beam is transported to the sample chamber after the pulse compression and then focused onto the sample via the off-axis parabolic (OAP) mirror with a focal length of 1.2 m (*f*/10) as shown in Fig. 1(*b*) ▸. The laser axis to the sample is about 73° off from the XFEL axis. The laser polarization is in the horizontal plane. The laser spot at the sample position is monitored with a 12-bit charge-coupled device (CCD) camera with a magnification of ∼4. For the focus optimization and the pointing adjustment to the position where the XFEL intersects with the sample plane, the laser pulse is amplified similarly to the experiments and then attenuated with low-reflectivity optics before the pulse compression. This allows compensating for the wavefront distortion in the Ti:sapphire crystals in the amplifiers. Fig. 4(*d*) ▸ shows an example of the focused beam of size 15 µm × 20 µm FWHM. Improvements of the beam profile at the focus are still ongoing, particularly to increase the encircled energy within the spot.

In early users’ experiments, started in 2018, the laser system was operated with a maximum energy of around 8 J after the pulse compression and with a pulse duration of 30–40 fs, resulting in a power of ∼200 TW. The parameters are determined to achieve reasonable shot-to-shot stability and for robust operation under the current status.

### 2.4. Dedicated instruments for experiments using the high-intensity laser   

For experiments using the XFEL and the high-intensity lasers, the instruments will be placed both inside and outside of the sample chamber. In addition to a sample mounting system, a couple of basic common instruments have been developed and installed to diagnose the high-intensity laser–matter interactions, such as detectors of the energetic particles and X-rays as shown in Fig. 1(*b*) ▸. Some basic diagnostics, such as for ions or optical measurements, have not been equipped yet. Users can bring their own instruments for the experiments. Viewports with an opening diameter of over 100 mm may be used for the optical measurements.

A motorized mounting system is prepared for solid samples. The system is equipped with six-axes stages for the sample alignment (three translation stages and three rotation stages) and with an additional rotation stage to change plates of the sample holder. Several sample plates are fixed on the sample mounting system, where a single plate typically contains 20–30 samples so that over 100 samples can be loaded at once to prevent frequent openings of the sample chamber [inset of Fig. 1(*b*) ▸]. Note that it takes 2–3 h typically to evacuate the sample chamber from the atmosphere to the vacuum pressure below 5 × 10^−3^ Pa, which is necessary for high-power laser shots. The sample shot rate is mainly limited due to the sample delivery and its alignment under current experimental conditions. Another possibility to limit the shot repetition rate is the radiation safety issue, particularly when the effective laser intensity becomes much higher. The radiation is monitored in and out of the experimental hutch to estimate the allowable shot rate with support from numerical simulations.

A flat crystal X-ray spectrometer measuring the spectra of emitted X-rays from the laser-irradiated matter is designed to work with either a highly oriented pyrolytic graphite (HOPG) or a pentaerythritol (PET) crystal. Here, the HOPG of ZYA grade has a mosaic spread of 0.4°. The dispersed X-rays are captured with an MPCCD detector (512 × 1024 pixels, 50 µm pixel^−1^, 16 bit data depth) (Kameshima *et al.*, 2014 ▸). In the top view, the distance from the sample to the crystal is ∼50 cm, which is equal to the distance from the crystal to the detector (1:1 magnification geometry). The crystal sizes are 26 mm × 150 mm and 20 mm × 100 mm for the PET and the HOPG, respectively. The crystal is placed below the beam height. The crystal height can be adjusted with a motorized stage to change the detectable X-ray energies. The lower limit of the detectable energies is ∼6 keV and the upper limit is ∼15 keV.

Note that the spectral window in a single-shot measurement depends on the crystal type and its height. For example, Cu *K*_α_ (8.05 keV) and *K*_β_ (8.9 keV) X-rays can be in the spectral window simultaneously either with the HOPG or the PET crystal with an energy dispersion of 2–3 eV pixel^−1^.

A vacuum electron spectrometer using a pair of permanent magnets has been installed on the laser axis. The solid angle of the spectrometer is limited with a 1 cm-thick collimator made of heavy metal. A scintillating screen, DRZ-High (Mitsubishi Chemical Co. Ltd) (Nakanii *et al.*, 2015 ▸), is placed at the exit of the magnet and covered with a thin aluminium foil to minimize the influence of the scattered laser light. The light emitted from the rear surface of the screen is observed with a 12 bit CCD to record the electron energy profiles. The detectable energy range can be selected from up to 20 MeV or up to 40 MeV by using a magnet with a maximum field strength of 0.2 T or 0.4 T, respectively. The energy dispersion on the screen is ∼0.6 MeV mm^−1^ or better for the case with a 0.4 T magnet. Here, the calibration of the spectrometer is essential to obtain quantitative information. In particular, the relation between the electron number on the scintillating screen and the signal intensity recorded with the camera is directly influenced by the imaging optics; therefore, it is important to execute the calibration on-site. Since the developed spectrometer can also be operated with an imaging plate (IP), which has been absolutely calibrated (Tanaka *et al.*, 2005 ▸; Chen *et al.*, 2008 ▸), the cross-calibration will be performed in comparison with IPs.

## 3. Demonstrations   

### 3.1. Laser–matter interactions with the high-intensity laser   

For commissioning of the experimental platform, the high-intensity laser has been used to irradiate samples and the interactions have been diagnosed with developed instruments as a demonstration of the platform. In this section, an example is presented from the test shots carried out with the east beam of the high-intensity laser system. The energy and the pulse duration after the compression are 8 J and 40 fs, respectively; thus the laser power is 200 TW. The laser is focused on a 20 µm-thick Cu foil with an incident angle of 45° in *p*-polarization. The focused intensity is estimated to be 7 × 10^18^ W cm^−2^ taking into account the encircled energy within the spot.

**Figure 5:**
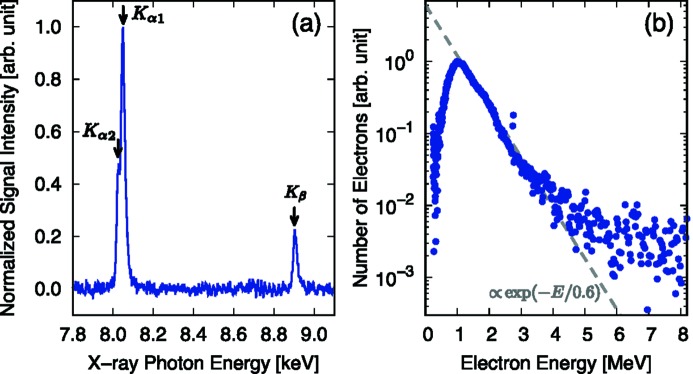
Results observed in a demonstration shot of the high-intensity laser with a power of 200 TW focused on a 20 µm-thick Cu foil. (*a*) X-ray spectrum showing the emission of Cu *K*_α_ and *K*_β_ X-rays. (*b*) Electron energy spectrum (dots) with an exponential fit showing the slope temperature of 0.6 MeV (dashed line).

The *K*-shell X-ray emission from the laser-irradiated Cu foil has been measured with the X-ray spectrometer using the HOPG crystal. A thin foil of Cu (20 µm thick) is placed in front of the MPCCD detector to reduce the background from the low-energy X-rays produced in the laser–matter interactions. The viewing axis of the spectrometer is ∼54° off from the target surface normal. The observed spectrum is shown in Fig. 5(*a*) ▸. The spectrum is normalized by the peak intensity of the *K*_α_ X-rays after the background subtraction. A good signal-to-noise ratio is achieved thanks to the high reflectivity of the HOPG crystal and an appropriate shielding of the spectrometer.

The spectral resolution of the developed spectrometer is evaluated based on the spectral width of the Cu *K*_β_ signal. The observed *K*_β_ signal has a width of ∼25 eV FWHM. Since the width is a convolution of the source profile and the instrumental broadening, the resolution is estimated to be Δ*E*/*E* ≃ 24/8900 ≃ 3 × 10^−3^ assuming the spectral width of the *K*_β_ line to be 5.9 eV (Hölzer *et al.*, 1997 ▸). Even though the source broadening effect is not taken into account in this discussion and the instrumental resolution could be better, the estimated resolution is consistent such that the *K*_α_ doublet structure (8.048 keV and 8.028 keV) is barely observed in Fig. 5(*a*) ▸.

The energy spectrum of energetic electrons accelerated via the laser–matter interactions are measured on the laser axis at ∼0.6 m from the sample through a 2 mm-diameter pinhole in front of the magnet with 0.2 T field strength. The emission from the scintillator (DRZ-High) is captured by a CCD camera, with a spatial resolution of 0.13 mm pixel^−1^. The spectral resolution is limited by the source size that is equal to the pinhole diameter at the spectrometer entrance. The resolution is estimated as Δ*E*/*E* ≃ 5 × 10^−2^ for 1 MeV electrons. The error becomes larger as the electron energy increases and almost saturates as Δ*E*/*E* ≃ 1 × 10^−1^ when the energy is above 5 MeV. An exponential fit of the spectrum yields a slope temperature of 0.6 MeV [Fig. 5(*b*) ▸], which is consistent with the temperature estimated from the laser intensity based on the ponderomotive scaling (0.5 MeV for 7 × 10^18^ W cm^−2^ with a laser wavelength of 800 nm) (Wilks *et al.*, 1992 ▸). In the analysis, the relative sensitivities of DRZ-High are assumed to be constant (Masuda *et al.*, 2008 ▸) in the energy range observed on the shot, which might not be an appropriate assumption specifically for the electrons below 1 MeV. In addition, the absolute calibration has not been performed under the current setup. Note that the absolute number of electrons is not required to estimate the slope temperature, and the spectrum below 1 MeV does not have much of an affect on the temperature estimation.

In high-power, high-intensity laser experiments, strong electromagnetic pulses (EMPs) can be a common issue that may cause problems or damage the instruments. So far, at least under the current experimental conditions, we have not experienced any issues at the experimental platform caused by EMPs. Here, the target is mounted on a metallic holder as shown in the inset of Fig. 1(*b*) ▸ and not electrically decoupled from the rest of the sample chamber.

### 3.2. Overlap of the XFEL and high-intensity laser   

In addition to the demonstration just using the optical laser at high intensities, the spatial and temporal overlaps have been examined using the XFEL and the high-intensity laser simultaneously. In this section, the general overlapping procedures and performance are described.

The spatial overlap between the XFEL and the high-intensity laser is adjusted via two steps. In the first step, the XFEL beam position is confirmed with cross-wires in the three-dimensional (3D) space by performing wire scans in 2D space. Here, the position in the extra one-dimensional (1D) direction, which is along the XFEL axis, is determined by the focusing monitor of the high-intensity laser. After the first step is completed, the XFEL position is recorded on the focusing monitor, which determines the intersection of the axes of the XFEL and the focusing monitor. Since the axis of the focusing monitor should be the same as the axis of the high-intensity laser from the focusing mirror to the sample, once the laser focus is adjusted to the position determined on the monitor the pointing of the laser is aligned on the XFEL axis, which is the second step.

The spatial overlaps have been tested as follows. A thin metal foil is placed at the intersection of the two beams, then low-energy pulses of both the XFEL and the optical laser are focused on the sample. The energy, especially of the optical laser, is adjusted so that the sample is slightly damaged but not completely destroyed. The damage created by the two beams is observed with a microscope to estimate the error of the spatial overlap, which is determined as a deviation between the centers of two areas of damage. The observed deviations are up to ∼50 µm. Here, the deviations can be caused by the combination of a pointing fluctuation of the optical laser and also an alignment error of the sample foil. The pointing fluctuation of the XFEL is smaller than the focal spot, therefore the influence is negligible for the spatial overlap currently. Regarding the optical laser pointing stability, the peak-to-peak fluctuation of ∼50 µm has been observed during the focus optimization of the optical laser. Note that the average position of the laser pointing is adjusted to overlap with the XFEL beam during the second step in the spatial overlap procedures. The alignment error of the sample foil can also cause a deviation of the two beams on the sample. The procedure and the monitors that we have used to align the sample at the interaction point could have an error of a few tens of micrometres in the direction. In our experimental configuration, for example, the irradiation points of the XFEL and the optical laser are 40 µm away if the foil is misaligned by 20 µm in the perpendicular direction to the sample surface. The deviations up to ∼50 µm can be explained as a result of the combination of these factors. Improvements of the laser pointing fluctuations as well as the target monitoring system are crucial for achieving spatial overlap more precisely.

The adjustment of two beams (XFEL and optical laser) in time at the sample position has been performed with ∼100 fs accuracy using a GaAs wafer. As detailed elsewhere (Sato *et al.*, 2015 ▸; Katayama *et al.*, 2016 ▸), the density of free carriers (electron and holes) in GaAs is changed by the irradiation of the XFEL, and then the optical transmission rapidly decreases for the light with photon energies above 1.43 eV or wavelengths shorter than 867 nm. The transmission of the laser pulse from the high-intensity laser system (wavelength of 800 nm) through the GaAs wafer is monitored in the timing adjustment procedure. In the process, the GaAs wafer is placed at the sample position perpendicular to the axis of the high-intensity laser. The optical laser is adjusted to have a large enough spot so that either the focused or unfocused XFEL beam overlaps within a smooth beam spot of the optical laser on the GaAs wafer. The optical images of transmitted laser light, which are observed with the laser focus monitor, show a clear change of the transmission only in the XFEL spot when the optical laser arrives at the GaAs wafer after the XFEL. The delay of the optical laser is scanned to find the beginning of the transmission change, which is defined as time zero.

**Figure 6:**
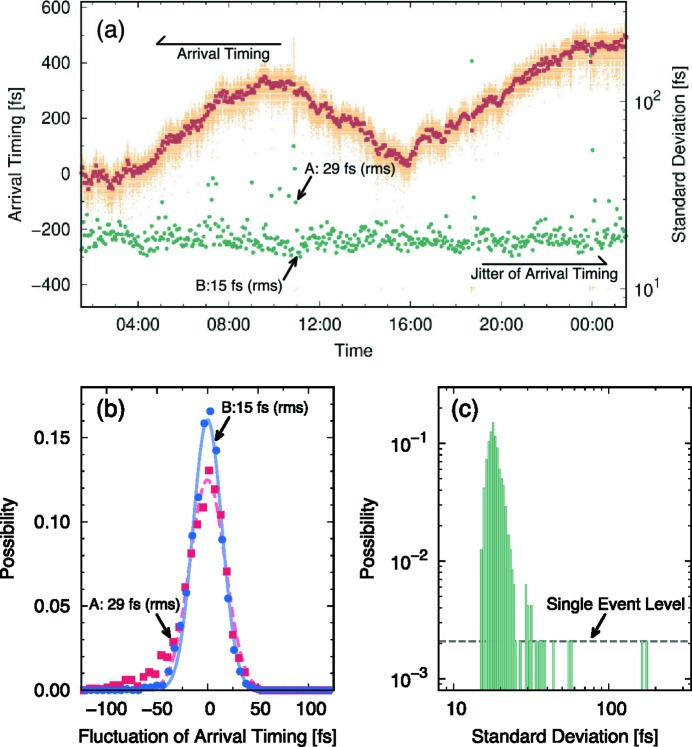
(*a*) Trends of the arrival timing measured for 24 h. The raw data of the relative arrival timing (light orange) is used to estimate its moving average (dark orange) and the standard deviation in r.m.s. (green) every 3 min. (*b*) Histograms of the relative arrival timing accumulated in 3 min. Two data sets shown with red squares (data A) and blue dots (data B) are observed at the time indicated in (*a*). Each result is fitted with a Gaussian profile. (*c*) Histogram of the jitter (standard deviations) in 24 h. The dashed line represents the single event level.

Even after the timing adjustment, the relative arrival timing of the optical laser to the XFEL can fluctuate at the sample position. Fluctuations both in a short period (jitter) and in a long period (drift) have a direct impact on the experimental observations. The fluctuations of arrival timing have been investigated using a similar method that has been applied to a timing tool in BL3 with the synchronized laser system (Katayama *et al.*, 2016 ▸). In the method, the XFEL is slightly focused with CRLs in 2D and makes a smooth footprint on the GaAs wafer covering the view area of the focus monitor. Since the incident angle of the XFEL to the GaAs wafer is 73°, the arrival timing of the XFEL depends on the position in the horizontal direction. Therefore, the fluctuation of the arrival timing appears as a horizontal shift of the position where the transmission changes, which is called the spatial decoding detection of the arrival timing fluctuation. With this technique, the relative timing fluctuations between the 10 keV XFEL and the high-intensity laser at the sample position have been measured for 24 h at a 10 Hz repetition rate. Software developed for the timing tool at SACLA (Nakajima *et al.*, 2018 ▸) is used for the data analysis. Note that the correction factor of the space and the time has been confirmed as 5.9 fs pixel^−1^, which is consistent with the optical geometry and the monitoring system used in the experiment. The results are summarized in Fig. 6 ▸. The timing fluctuation in 3 min (*i.e.* 1800 events), namely the jitter, is ∼20 fs r.m.s., which is shorter than the pulse duration of the high-intensity laser. Histograms of the arrival timing in the short period are fitted well with a Gaussian distribution when the jitter is small as shown in Fig. 6(*b*) ▸. In contrast, some scattered distributions are observed for relatively large jitter cases. The jitter is almost constant for 24 h; however, significant large jitters are also observed occasionally as one can see in Fig. 6(*c*) ▸, which is a histogram of the jitter for 24 h. Since most of the large jitters are observed just after the accelerator trip (interlocked stop), this is likely due to the after effect of the trip. Here, the accelerator trip is caused by the RF breakdown, the pre-firing of the thyratrons, and the arcing in the klystrons (Inagaki *et al.*, 2014 ▸). The jitter becomes normal typically in 10–15 min after the trip.

More severe fluctuations from a few hundreds of femto­seconds to half a picosecond are observed in the long-term (over an hour) period as shown in Fig. 6(*a*) ▸. The envelope of such slow fluctuations has a gradient of 50–70 fs h^−1^. In the early phase of the commissioning of the platform, we had observed a much larger drift (>ps h^−1^) in similar measurements. There had been a clear correlation between the temporal evolutions of the arrival timing and the room temperature of LH6. The temperature fluctuation has been stabilized to ±0.1°C thereafter, and then the direct correlation is not seen between the arrival timing and the room temperature under the current condition. The source of the drift will be investigated further.

## 4. Conclusion and perspectives   

The combination of XFELs and high-intensity lasers brings novel capabilities to experimental research in HED science. The current status and the capabilities are summarized on the experimental platform using a high-intensity laser in combination with a hard XFEL at SACLA, where early users’ experiments have been performed with a laser power of 200 TW since 2018. For the combinative use of the two types of lasers, the light source performance has been characterized during the commissioning of the experimental platform. The dedicated diagnostics for the platform have also been developed and confirmed that they have functioned properly with the XFEL and the high-intensity laser. Synchronizations in space and time have been demonstrated and planned to be improved for more precise experiments. Further development and optimization will be conducted particularly for the stable and robust operation and to maximize the capabilities of the experimental platform.
